# Supplementary material for: Interactive System for Similarity-Based Inspection and Assessment of the Well-Being of mHealth Users
Source: Entropy (Basel). 2021 Dec 17;23(12):1695. doi: 10.3390/e23121695 (PMC8700569; doi:10.3390/e23121695)
Supplement: Supplementary file 1 [file entropy-23-01695-s001.zip › entropy-1458252-supplementary.pdf]

# Supplementary Materials:

**Subash Prakash [1], Vishnu Unnikrishnan [1], Rüdiger Pryss [2], Robin Kraft [3], Johannes Schobel [4], Ronny Hannemann [5], Berthold Langguth [6], Winfried Schlee [6], Myra Spiliopoulou [1]**

## 1. Comparison over the similarity constructed through Loudness-s02

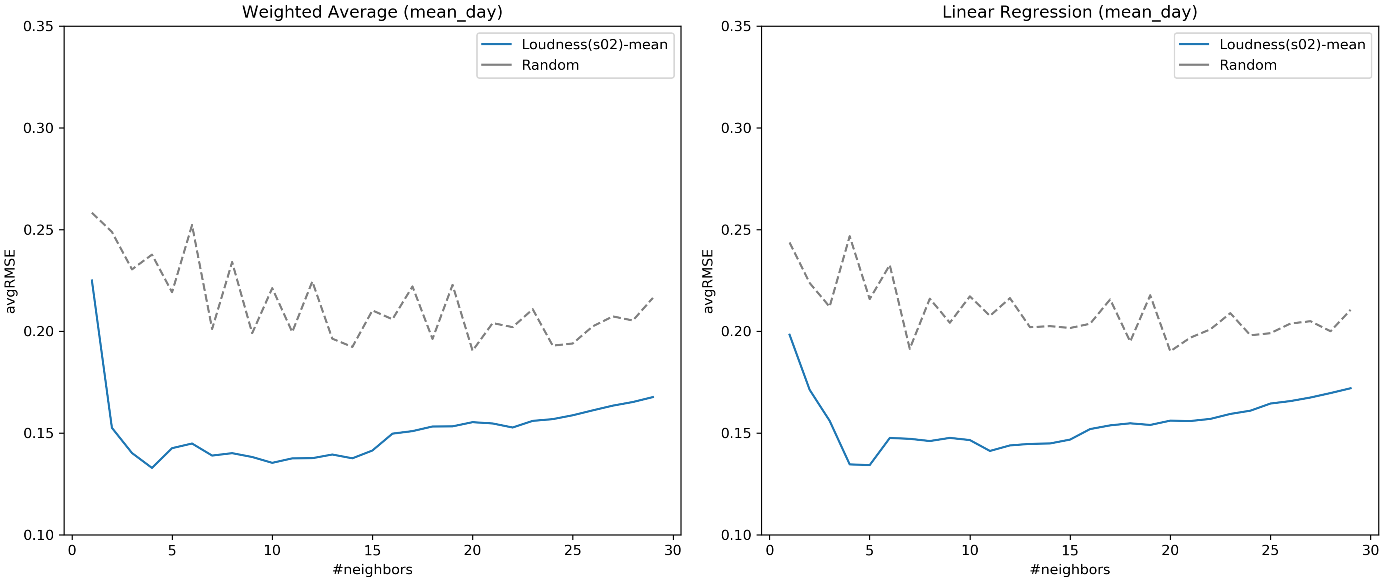


**Figure S1.** RMSE over Loudness-(s02)

## 2. RQ2:User Outlierness

The two reference (termed as Ref) users who have differences in their tinnitus loudness and distress observations are shown:

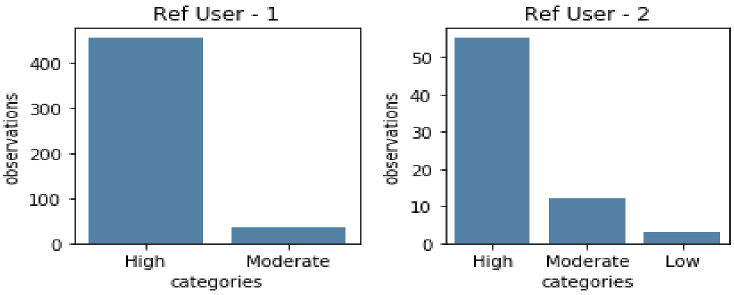


**Figure S2.** User's recordings distributed across the categories for loudness(s02)

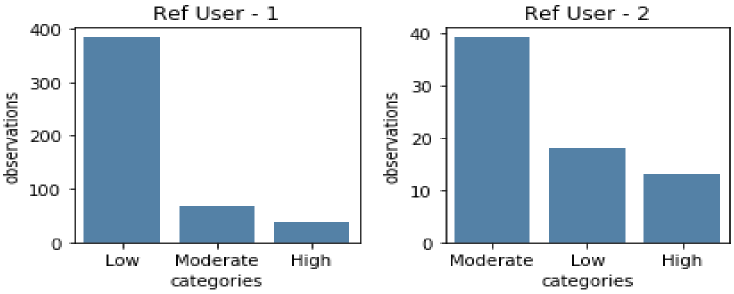


**Figure S3.** User's recordings distributed across the categories for tinnitus distress(s03)
